# Tinnitus is associated with increased extracellular matrix density in the auditory cortex of Mongolian gerbils

**DOI:** 10.1186/s12868-024-00904-w

**Published:** 2024-10-17

**Authors:** Konstantin Tziridis, Antonia Maul, Jwan Rasheed, Patrick Krauss, Achim Schilling, Holger Schulze

**Affiliations:** 1 Experimental Otolaryngology, Head and Neck Surgery, University Hospital Erlangen, ENT Hospital, Waldstrasse 1, 91054 Erlangen, Germany; 2 Friedrich-Alexander University Erlangen-Nürnberg, CCN group, pattern recognition lab, Immerwahrstrasse 2A, 91058 Erlangen, Germany

**Keywords:** Tinnitus, Extracellular matrix, Auditory cortex, Animal model, Rodents

## Abstract

Most scientists agree that subjective tinnitus is the pathological result of an interaction of damage to the peripheral auditory system and central neuroplastic adaptations. Here we investigate such tinnitus related adaptations in the primary auditory cortex (AC) 7 and 13 days after noise trauma induction of tinnitus by quantifying the density of the extracellular matrix (ECM) in the AC of Mongolian gerbils (*Meriones unguiculatus*). The ECM density has been shown to be relevant for neuroplastic processes and synaptic stability within the cortex. We utilized a mild monaural acoustic noise trauma in overall 22 gerbils to induce tinnitus and a sham exposure in 16 control (C) animals. Tinnitus was assessed by a behavioral response paradigm. Animals were separated for a presence (T) or absence (NT) of a tinnitus percept by a behavioral task. The ECM density 7 and 13 days after trauma was quantified using immunofluorescence luminance of Wisteria floribunda lectin-fluoresceine-5-isothiocyanate (WFA-FITC) on histological slices of the primary AC, relative to the non-auditory brainstem as a reference area. At both timepoints, we found that the WFA-FITC luminance of the AC of NT animals was not significantly different from that of C animals. However, we found a significant increase of luminance in T animals’ ACs compared to NT or C animals’ cortices. This effect was found exclusively on the AC side contralateral to the trauma ear. These results point to a hemisphere specific process of stabilization of synaptic connections in primary AC, which may be involved in the chronic manifestation of tinnitus.

## Introduction

Tinnitus—the perception of a sound without external physical source—is commonly believed to be the result of an interaction of a damage to the peripheral auditory system and central neuroplastic adaptations to the new, changed auditory input [40]. We already put forward a bottom-up model for tinnitus development in which these neuroplastic adaptations—after a mild hearing loss—start in the dorsal cochlear nuclei (DCN) after losing a part of the neuronal information not transmitted from the damaged cochlea anymore [29, 31, 54]. In a nutshell, this Erlangen model of tinnitus development [57] assumes that a neurophysiological mechanism detects a drop in information after a hearing loss by computing the autocorrelation of the signal of the cochlear nerve [58], as less temporally structured spike trains reach the DCN neurons [58]. By disinhibition of neuronal noise, most probably coming from the somatosensory system [54], a reduced—but still information containing—input signal plus that noise can reach the threshold and activate the DCN neurons, a mechanism known as stochastic resonance. As a consequence, otherwise sub-threshold activity from the cochlea can now be further transmitted along the auditory pathway, thereby optimizing information transmission within the auditory system. This comes at the cost of co-propagation of the noise up to the auditory cortex, where the activity is then perceived as a sound, namely tinnitus. based on that model we were able to predict and explain several tinnitus related effects on hearing loss, e.g., that the hearing thresholds in patients with mild to moderate hearing loss and tinnitus are better than in matched patients without tinnitus [16]. Furthermore, we were able to develop a new therapeutic approach based on that model which allows a causative reduction of tinnitus loudness in tinnitus patients [52, 63]. However, the original model failed to describe the effects of chronic manifestation of tinnitus and made no predictions on tinnitus related plasticity in the auditory cortex. To overcome these drawbacks, in a previous paper, the stochastic resonance model was unified with the predictive coding model of auditory phantom perception [53, 59]. in this view, tinnitus might be induced and chronically manifested through an interplay of two feedback loops—the stochastic resonance loop in the brainstem and the predictive coding circuit in the cortex [53, 59].

In the present study, we aim to investigate tinnitus related adaptations in the auditory cortex (AC) after an assumed chronic manifestation of the phantom percept, that could start as early as 1 week after an acoustic trauma [2]. Based on electrophysiological recordings in animals [13, 62] and e.g., imaging methods or EEG recordings in humans [19, 56], it is already known that tinnitus before and after chronification has different neurophysiological manifestations within the AC of mammals. While before chronification the neurophysiology of the AC seems to undergo profound changes in, e.g., tonotopy (e.g., [9]), the normal processing in the AC seems to be recovered after the new percept is developed, with the addition of the phantom sound activity in the affected frequency range [2, 34].

Neuronal plasticity in the cortex can be assessed using several different methods [23, 38, 43, 46]. One of them is the investigation of the density of the extracellular matrix (ECM) by using immunofluorescence luminance of Wisteria floribunda lectin-fluoresceine-5-isothiocyanate (WFA-FITC) on histological slices (e.g., [21]). The higher the density (WFA-FITC luminance) of the ECM, the more stable are the synaptic connections and therefore the less likely it is that a once formed connectivity pattern can be changed by new information [8, 18]. With that in mind, we asked, if and how the ECM density of the primary AC—and indirectly the potential for neuroplasticity of that area—is affected by tinnitus after its chronification in our animal model, the Mongolian gerbil (*Meriones unguiculatus*). To answer this question, we measured ECM density at two separate timepoints, 7 and 13 days after an acoustic trauma, where plasticity induced by tinnitus development seems to be completed [2].

## Methods

### Animals, housing and ethics statement

38 male Mongolian gerbils, purchased from Janvier (Le Genest-Saint-Isle, France) were housed in standard animal racks (Bio A.S. Vent Light, Zoonlab, Emmendingen, Germany) in groups of 3–4 animals with free access to water and food at a room temperature of 20–25 °C under a 12 h/12 h dark/light circle. At the beginning of the experiments, the animals were 12 weeks old and had been at least 2 weeks in the animal facility for habituation. The use and care of the animals was approved by the state of Bavaria (Regierungspräsidium Mittelfranken, Ansbach, Germany, No. 54-2532.1-02/13) and (Regierungspräsidium Unterfranken, Würzburg, Germany, No. 55.2.2-2532-2-540).

### Experimental protocol

The animals were investigated twice with the behavioral paradigm of the gap prepulse inhibition of the acoustic startle response (GPIAS) to evaluate possible behavioral indications of a tinnitus percept and with auditory brainstem response (ABR) audiometry under anesthesia to obtain the hearing thresholds. The respective first measurements were performed 5 days before a monaural acoustic noise trauma (2 kHz, 115 dB SPL, 75 min; anesthetized) or sham trauma (2 kHz, 65 dB SPL, 75 min; anesthetized). The second ABR measurements were obtained 4 days after that trauma, and GPIAS was obtained immediately before sacrificing the animals (CO_2_ euthanasia) 7 days (N = 26; 13 noise trauma, 13 sham trauma) or 13 days (N = 12; 9 noise trauma, 3 sham trauma) after the trauma. The brains of the sacrificed animals were extracted, cryo-sliced, immunostained and immunohistologically evaluated. With the exception of the immunohistological analysis of the brain slices, all methods were used as described earlier (e.g., [55, 64, 66]).

### Behavioral measurements

For the GIPAS measurements for tinnitus assessment, a custom-made open-source setup was used as detailed in [15]. In short, the animals were placed in a wire mesh closed acrylic glass restrainer tube and placed on a 3D-acceleration sensor platform fixed to a vibration-damped table. Two loudspeakers in front of the animals at a distance of 10 cm presented the 115 dB SPL startle stimulus (Neo 25 S, SinusLive, noise burst 20 ms, flattened with 5 ms sin^2^ ramps) and the 60 dB SPL spectral noise background (CantonPlus XS.2). Spectral noise was centered at 1–16 kHz in octave steps with one octave bandwidth, with and without a gap of silence of 50 ms (flanked by 20 ms sin^2^ ramps, 10 ms complete silence) starting 100 ms before the startle stimulus.

Before measurements, animals were given 15 min of habituation in darkness in the tube. five habituation stimuli were presented prior to the actual measurement to “level” the startle responses. Each stimulus was repeated 30 times (15 with and 15 without gap), summing up to 120 stimuli, which took roughly 30 min.

The GPIAS effect was quantified by calculating the mean from the full combinatorial log-normalized startle amplitudes as a response to gap and no gap pre-stimulus with a custom-made Python program (for details see [51]). Statistics on the mean GPIAS results were performed with Statistica 14 (TIBCO Software GmbH, Munich, Germany; cf. below).

### Brainstem audiometry

The animals were anesthetized with a ketamine-xylocaine solution (ketamine 500 mg/kg, xylazine 25 mg/kg) for the frequency-specific ABR recordings. They were placed on a remote-controlled heating pad set to 37 °C. Three silver wires were used as electrodes and were placed subcutaneously retroaural above the bulla of the tested ear (recording electrode), central between both ears (reference electrode) and at the basis of the tail (ground electrode). The signal was differentially recorded between recording and reference electrode and filtered (bandpass filter 400–2000 Hz) via a Neuroamp 401 amplifier (JHM, Mainaschaff, Germany). Individual audiograms of both ears (duration around 30 min each) were obtained for stimulation frequencies between 1 and 16 kHz in octave steps for stimulation intensities ranging from 0 to 90 dB SPL in 5 dB steps (6 ms duration with 2 ms sine-square ramps) with 300 repetitions each.

### Monaural acoustic noise and sham trauma

Animals were put under deep ketamine-xylocaine anesthesia (cf. above). One ear (pseudorandomly selected) was plugged with foam (3 M^™^ earplugs 1110, 3 M, Neuss, Germany) adding at least 20 dB attenuation in the given frequency range [60]. The animals were placed on a remote-controlled heating pad set to 37 °C with the non-plugged ear towards a speaker (CantonPlus XS 2). The acoustic noise trauma (2 kHz, 115 dB SPL, 75 min) or sham trauma (2 kHz, 65 dB SPL, 75 min) was only performed on that ear.

### Brain extraction, cryo-slicing and immunofluorescence staining

The sacrificed animals were decapitated and the brains were extracted carefully. These were placed in a 2% paraformaldehyde / 2% sucrose solution on a rocking shaker for 8 h at 4 °C. The tissue was then washed with phosphate-buffered saline (PBS) three times over one minute each, and then transferred into a 10% sucrose solution on a rocking shaker for 8 h at 4 °C. The incubation was finalized with 15% sucrose solution and finally 30% sucrose solution on a rocking shaker for 8 h each at 4 °C, washing was performed with PBS two times for one minute each. After drying, the tissue was shock-frosted in liquid nitrogen at −196 °C and cur on a cryotome in 18 µm thick slices, which were transferred onto object slides and air-dried for 30 min at ambient temperature. Until staining, the slides were stored at −20 °C in a fridge,

After defrosting and drying, every 10th object slide was used and fixated with −20 °C cold methanol for three minutes and dried for 10 min. After that the slices were permeabilized with 2% horse-serum in 0.1% Triton X-100 in PBS solution over 2.5 h. Then the incubation with Wisteria Floribunda Lectin-Fluorescein-5-isothiocyanate (WFA-FITC) started, with WFA-FITC 1:200 in 0.5% horse-serum/PBS over 5 h at room temperature. It was stopped with washing with PBS three times over five minutes each. Finally, the slides were mounted with IS Mounting-Medium PI.

### Microscopy and densitometry

Immunostained tissue slices were investigated with a BZ 9000 Keyence fluorescence microscope (4 × magnification) and single pictures were combined to a full slice picture with the BZ-II software. We used a 1360 × 1024 pixel resolution, green fluorescence, exposure adjustments: shadow 11, highlight 165, gamma 0.7, exposure time 1/1.1 s.

**Fig. 1:**
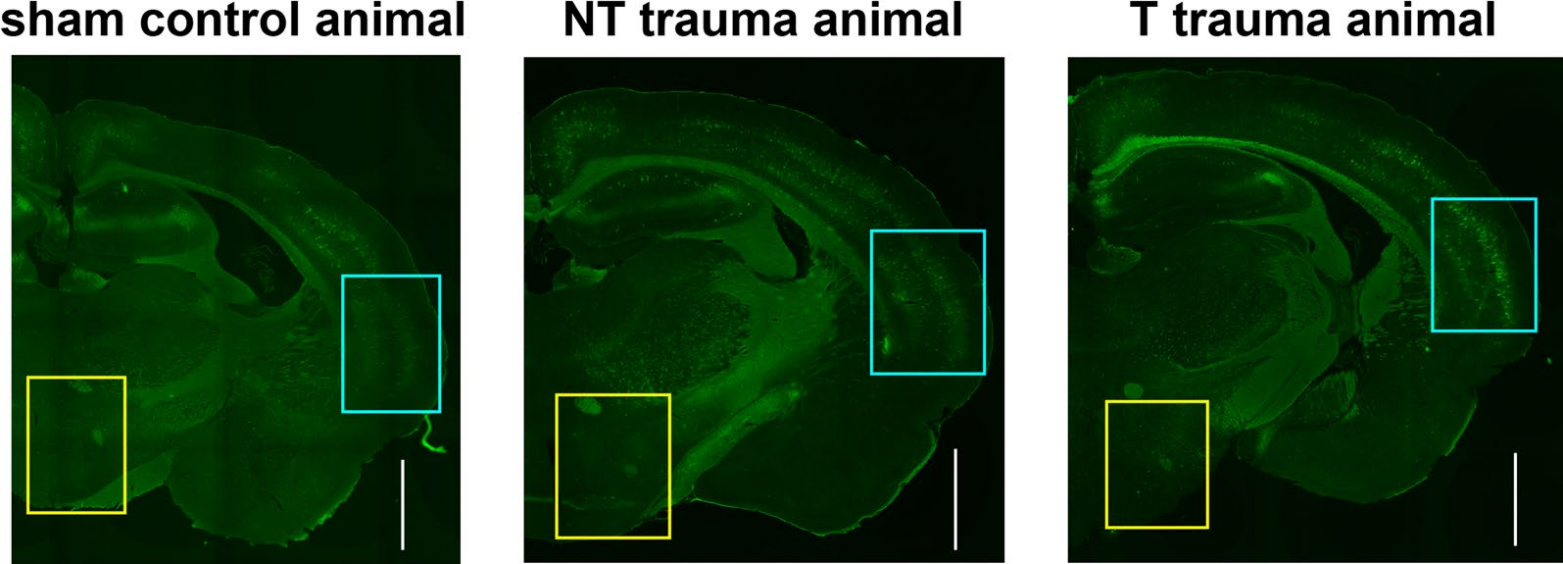
Exemplary WFA-FITC marked brain slices from a control (**left panel**), non-tinnitus (**center panel**) and tinnitus animal (**right panel**) 13 days after the trauma. Rectangles identify the evaluated cortical (turquoise)/brainstem reference (yellow) areas. White scale indicates 1 mm. Note that the luminance of the slices could vary, therefore only the ratio of cortex to brainstem luminance is used for further analyses

The combined slice-pictures were evaluated with ImageJ Fiji (version 1.53) and only the slices containing the primary auditory cortex were evaluated. For marker-intensity reference, always the brainstem of the same slide was used. The appropriate slices and cortical / subcortical areas were identified using anatomical markers based on a Mongolian gerbil brain atlas (plates 28–33 in [48]). The areas of interest ipsi and contralateral to the trauma ear were marked and their luminance (in pixel/mm^2^) was calculated using the inbuilt program function, similar to the densitometry described elsewhere (e.g., [7]). To ensure independence of any dye-related effects, the luminance values of the cortical areas were divided by values of reference brainstem area of the same size (i.e., luminance ratio). Examples of the brain slices of animals with trauma and behavioral signs of tinnitus, with trauma but without behavioral signs of tinnitus and sham trauma without tinnitus are depicted in Fig. 1.

### Statistics

As mentioned above, all statistics were performed with Statistica 14 for both timepoints independently. Tests for normal distributions were performed to ensure the validity of the performed analyses (e.g. ECM luminance ratio at 7 d and 13 d (c.f. below), Shapiro–Wilk test, W = 0.990, p = 0.91 and W = 0.991, p = 0.63).

The effect of the trauma on the hearing thresholds obtained with the ABR measurements was assessed using the calculated [49] individual and frequency specific hearing loss (HL, post threshold—pre threshold) with a two-factorial ANOVA (factor *ear* and *frequency*).

For the evaluation of the GPIAS results, the means of the effect size of the individual log-normalized gap / no-gap amplitude responses of the different background noise frequencies were analyzed for each animal and each frequency [51]. Significant negative effect sizes (Students t-tests) at a given frequency indicate a possible tinnitus percept and an animal with such indication was classified as a tinnitus animal (T group), if no such behavioral sign of tinnitus was found, the animal was classified as non-tinnitus animal (NT group). All sham-trauma receiving animals showed no signs of significant tinnitus and were assigned to the respective control group C at 7 or 13 days).

The luminance ratio of the ECM of the cortical areas was analyzed using two-factorial ANOVAs (factor *side* (ipsi- / contralateral to trauma) and *animal group* (T, NT, C)) independent for the timepoint of brain extraction. Additionally we investigated the luminance ratios of the two hemispheres separately by one-factorial ANOVAs (with Bonferroni correction for analysis repetition) with the factor *group* only. This was done to find possible side-specific effects that were obscured due to the variance in the comparably few data points of the overall 26 and 12 animals at the two timepoints.

## Results

### Hearing loss and tinnitus

As detailed in the Method section, the HL was assessed by individual ABR threshold comparison. HL group effects were analyzed using a two-factorial ANOVA with the factors *ear* (plugged control ear, trauma ear, sham trauma ear) and *frequency* (1 kHz, 2 kHz, 4 kHz, 8 kHz, 16 kHz).

In the 7 days group (ABR measurement 4 days after the trauma), the results of the factor *ear* (F(2, 110) = 4.57, p = 0.012) revealed a significant higher mean HL (± standard error) in the trauma ears (12.59 dB ± 3.39 dB) compared to the plugged control ears (-0.28 dB ± 2.61 dB, Tukey post-hoc test: p = 0.011) but only a tendency for a difference with the sham trauma ears (3.38 dB ± 3.02 dB, Tukey post-hoc test: p = 0.057). Plugged control and sham trauma ears did not show a significant difference in HL (Tukey post-hoc test: p = 0.82). Neither in the factor *frequency* (F(4, 110) = 2.29, p = 0.06) nor in the interaction of both factors (F(8, 110) = 0.95, p = 0.48) a significant effect on the HL was found. In other words, the traumatized ears showed a significant general (while not frequency specific) increase in hearing thresholds after the trauma. On the other hand, neither plugged control nor sham trauma ears were affected by the noise exposure.

**Fig. 2:**
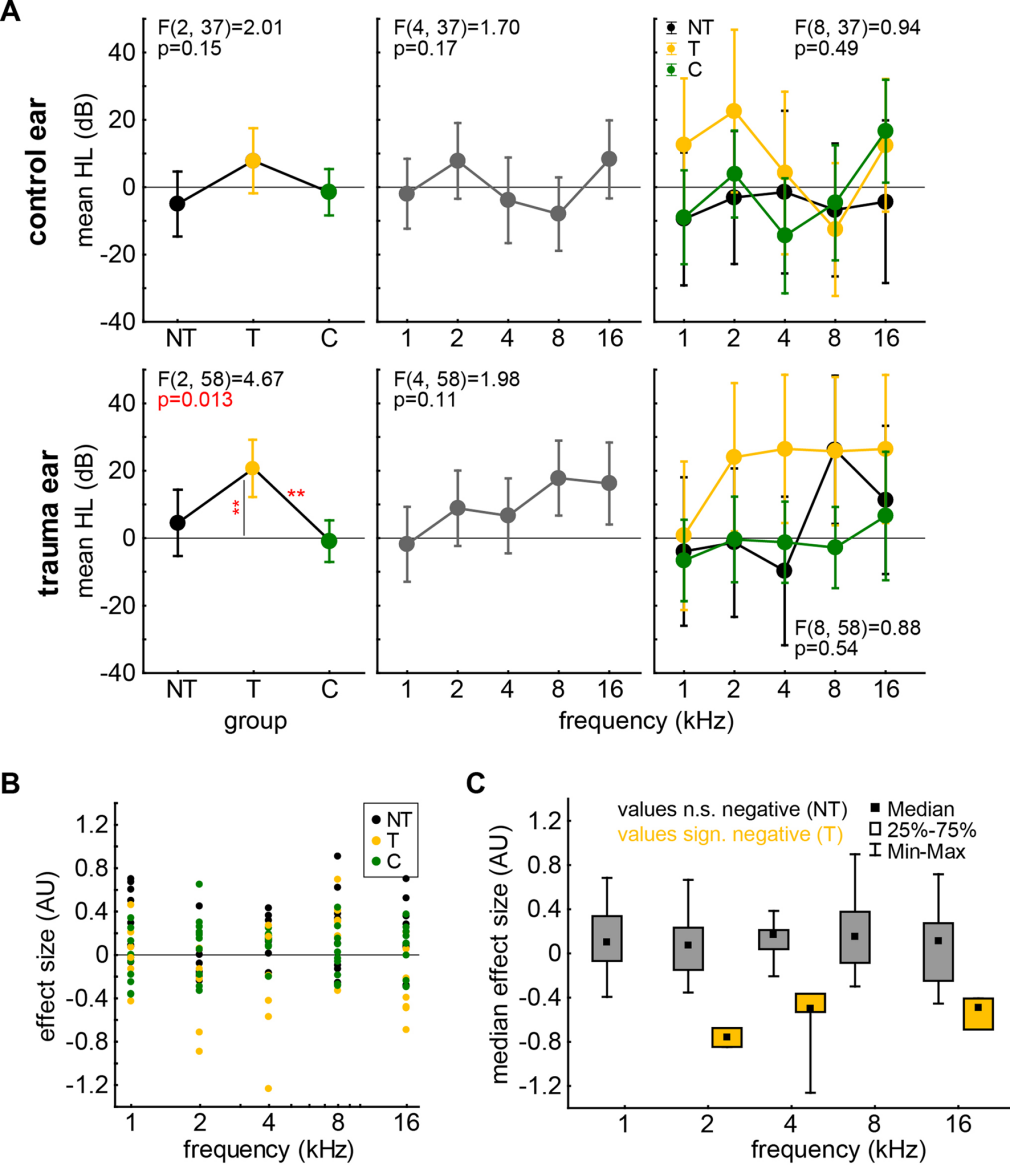
**A** 7 d after trauma results of 2-factorial ANOVAs of mean hearing loss (HL in dB) of plugged control ears (upper panels) and (sham-) trauma ears (lower panels) with the factors *group* (NT, T, C; color-coded) and *frequency* (in kHz). Asterisks above horizontal lines give Tukey post-hoc test values (**p < 0.01) and asterisks next to vertical lines give significant single sample t-test values against 0 (**p < 0.01). **B** Results of GPIAS tests (effect size in AU) for all animals in the three groups over the five tested frequencies. **C** Separation of non-significant (NT) and significantly negative (T) effect size values across the five tested frequencies

Of those 13 animals that received a monaural acoustic noise trauma, only four developed behavioral signs of a tinnitus percept (measurement 7 days after trauma) in at least one tested frequency (2 kHz, 4 kHz, 16 kHz; T group). This was indicated by a significant decrease in effect size in the GPIAS paradigm 7 d after the trauma (t-tests, p < 0.05). The other nine trauma animals (NT group) did not show any significant decrease in effect size. For the HL effects across those three animal groups in affected and plugged ears please refer to Fig. 2A. Out of the four T animals, one animals developed a possible tinnitus percept at two frequencies (4 kHz and 16 kHz), the other three animals at three frequencies (2 kHz, 4 kHz and 16 kHz). For an overview of the effect size values of all animals and the separation into T and NT values please see Fig. 2B, C.

For the 13 days group (ABR measurement 4 days after the trauma), the results of the 2-factorial ANOVA were very similar to the results described above. In the factor *ear* (F(2, 148) = 12.74, p < 0.001) a significant higher mean HL (± standard error) in the trauma ears (9.75 dB ± 1.26 dB) compared to the plugged control ears (2.01 dB ± 1.17 dB, Tukey post-hoc test: p < 0.001) and here also the sham trauma ears (−0.86 dB ± 2.69 dB, Tukey post-hoc test: p = 0.001) was revealed. Also here, the plugged control and sham trauma ears did not show a significant difference in HL (Tukey post-hoc test: p = 0.59). Again, neither in the factor *frequency* (F(4, 148) = 0.10, p = 0.98) nor in the interaction of both factors (F(8, 148) = 0.27, p = 0.98) we found a significant effect on the HL. In other words, as described for the 7 d group, the traumatized ears showed a general significant increase in hearing thresholds after the trauma while neither plugged control nor sham trauma ears were affected by the noise exposure.

**Fig. 3:**
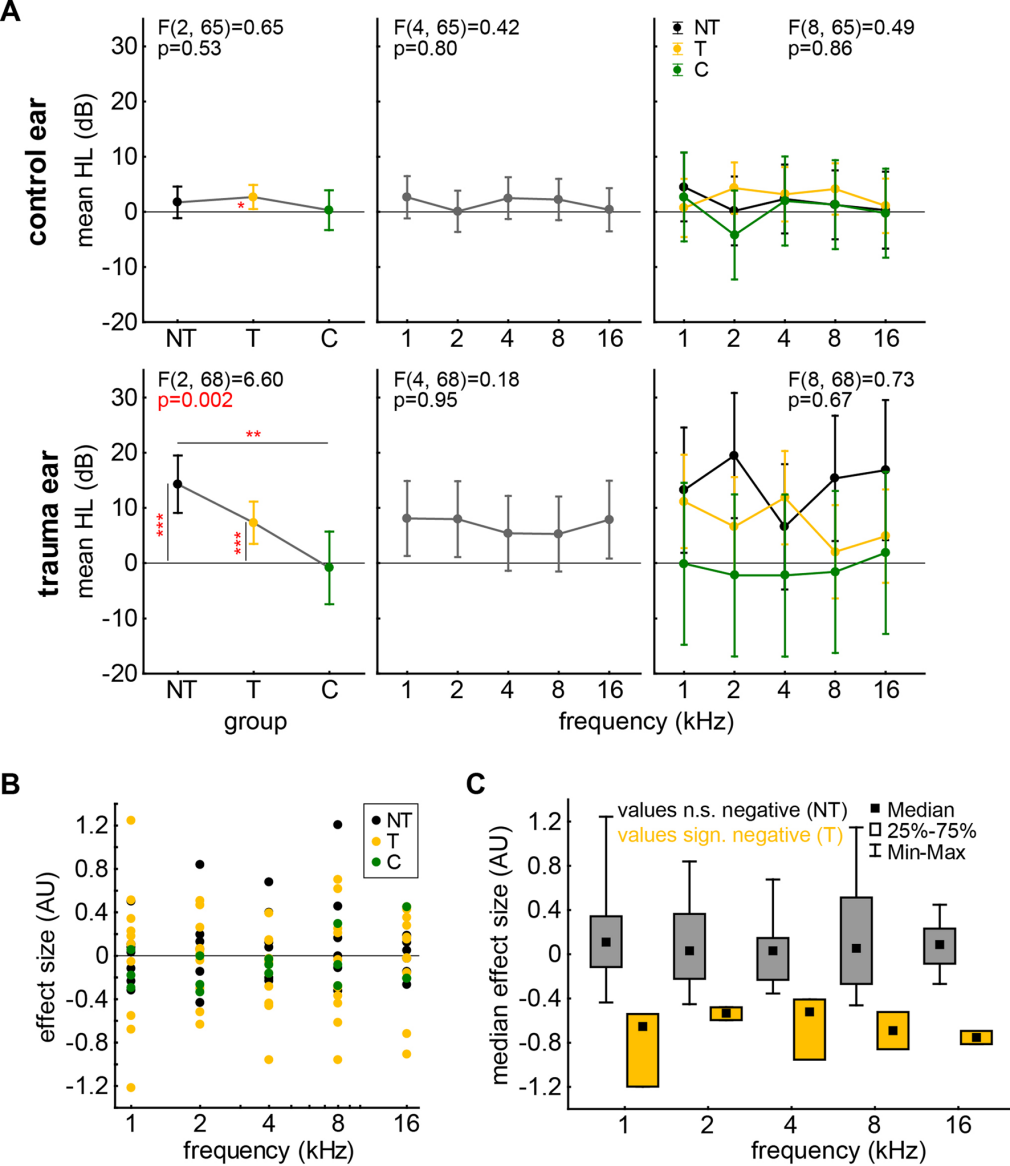
**A** 13 d after trauma results of 2-factorial ANOVAs of mean hearing loss (HL in dB) of plugged control ears (upper panels) and (sham-) trauma ears (lower panels) with the factors *group* (NT, T, C; color-coded) and *frequency* (in kHz). Asterisks above horizontal lines give Tukey post-hoc test values (**p < 0.01) and asterisks next to vertical lines give significant single sample t-test values against 0 (*p < 0.05; ***p < 0.001). **B** Results of GPIAS tests (effect size in AU) for all animals in the three groups over the five tested frequencies. **C** Separation of non-significant (NT) and significantly negative (T) effect size values across the five tested frequencies

As for the 7 days group, of the nine animals that received a monaural acoustic noise trauma in the 13 days group, four developed behavioral signs of a possible tinnitus percept (measurement 13 days after trauma) in at least one tested frequency (1 kHz, 2 kHz, 4 kHz, 8 kHz, 16 kHz; T group). As described above, this was indicated by a significant decrease in effect size in the GPIAS paradigm 13 d after the trauma (t-tests, p < 0.05). The other five trauma animals (NT group) as well as the three control animals (C group) did not show any significant decrease in effect size. For the HL effects across those three animal groups in affected and plugged ears please refer to Fig. 3A. Note that there NT animals’ trauma ears showed a somewhat higher mean HL than T animals’ trauma ears when compared to the control group (Tukey post-hoc tests: NT vs. C. p = 0.002; T vs C, p = 0.09). Out of the four T animals, two animals developed a possible tinnitus percept at one frequency (2 kHz or 4 kHz), two animal at two frequencies (4 kHz and 8 kHz or 1 kHz and 16 kHz) and one at three frequencies (1 kHz, 8 kHz and 16 kHz). For an overview of the effect size values of all animals and the separation into T and NT values please see Fig. 3B, C.

### Cortical ECM density

The luminance ratio of the cortex sides ipsi- and contralateral to the trauma or sham trauma ear was assessed in the T, NT and C groups with two-factorial ANOVA with the factors *side* and *group* independent for both timepoints.

Seven days after trauma we found (Fig. 2A) a significant *group* effect (F(2, 388) = 3.55, p = 0.030). The mean luminance ration of the T animals’ brains (0.87 ± 0.05) showed a tendency of being higher than that of the NT’s (0.75 ± 0.03, Tukey post-hoc tests: p = 0.07) but was significantly higher than the ratio in the sham control brains (0.73 ± 0.02, Tukey post-hoc tests: p = 0.022). Neither in the *cortex side* (F(1, 388) = 0.05, p = 0.83) nor in the *interaction* analyses (F(2, 388) = 1.68, p = 0.19) a significant effect could be found. Therefore, this first analysis points to a general increase in luminance ratio (*group* effect) and therefore in ECM density in the auditory cortex of animals with behavioral signs of tinnitus, at least relative to sham trauma animals.

**Fig. 4:**
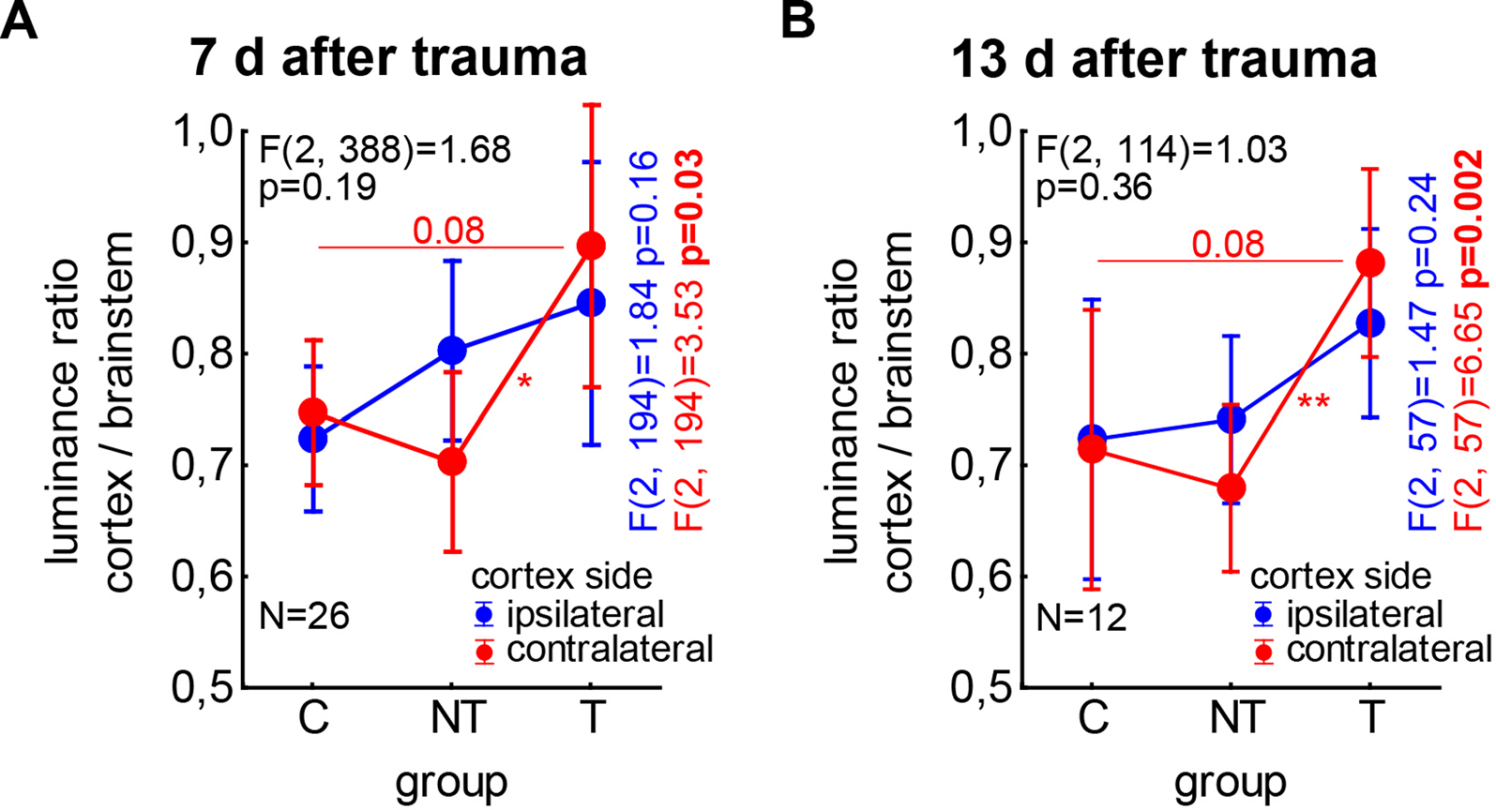
Interaction plots of the two-factorial ANOVAs of the normal distributed luminance ratio for the factors *animal group* and *cortex side* for both observation times separately. **A** The interaction on day 7 after trauma, the F statistics is given in black. The two colored F-statistics show the results of the Bonferroni corrected one-factorial ANOVAs of ipsi- (blue) and contralateral (red) luminance ratios of the three animal groups. **B** The interaction on day 13 after trauma, colors as above. Colored asterisks and numbers in both panels indicate the significance levels and tendencies of the Tukey post-hoc tests in the one-factorial ANOVAS: * p < 0.05, ** p < 0.01

When we investigated the two hemispheres separately (and corrected for repeated testing), we found the described significant ECM density (i.e., luminance ratio) increase in T animals only in the auditory cortex contralateral to the trauma (Fig. 4A, color-coded F-statistics): There, the one-factorial ANOVA (F(2, 194) = 3.53, p = 0.031) revealed a higher ECM density (T: 0.90 ± 0.06; NT: 0.70 ± 0.04; C: 0.75 ± 0.03) with a significant Tukey post-hoc test between T and NT with p = 0.022 and a tendency for a higher tinnitus related ECM density between T and C with p = 0.08; no difference was found between NT and C with p = 0.66. In the cortex side ipsilateral to the trauma, no significant differences (F(2, 194) = 1.84, p = 0.16) were found between the ECM density of the three animal groups (T: 0.85 ± 0.07; NT: 0.80 ± 0.04; C: 0.72 ± 0.03); also the Tukey post-hoc tests did not show any significant differences.

Similarly as in the 7 days group, 13 days after trauma (Fig. 4B) a significant *group* effect (F(2, 114) = 7.05, p = 0.001) with the mean luminance ration of the T animals’ brains (0.85 ± 0.03) being significantly higher than those of the NT (0.71 ± 0.03, Tukey post-hoc tests: p = 0.001) and sham control brains (0.72 ± 0.04, Tukey post-hoc tests: p = 0.035) could be identified. As before, neither in the *cortex side* (F(1, 114) = 0.02, p = 0.89) nor in the *interaction* analyses (F(2, 114) = 1.03, p = 0.36) a significant effect could be found. Also this analysis points to a general increase in luminance ratio and therefore in ECM density in the auditory cortex of animals with behavioral signs of tinnitus, this time relative to NT and sham control animals.

When we investigated the two hemispheres separately, we again found that the described significant ECM density increase in T animals could be found only in the auditory cortex contralateral to the trauma (Fig. 4B, color-coded F-statistics): There, the one-factorial ANOVA (F(2, 57) = 6.65, p = 0.002) revealed a higher ECM density (T: 0.88 ± 0.04; NT: 0.68 ± 0.04; C: 0.71 ± 0.06) with a significant Tukey post-hoc test between T and NT with p = 0.002 and a tendency for a higher tinnitus related ECM density between T and C with p = 0.08; no difference was found between NT and C with p = 0.88. As described for the 7 days group, the cortex side ipsilateral to the trauma showed no significant differences (F(2, 57) = 1.47, p = 0.24) between the ECM density of the three animal groups (T: 0.83 ± 0.04; NT: 0.74 ± 0.04; C: 0.72 ± 0.06); also the Tukey post-hoc tests did not show any significant differences.

Taken together, our results point to an increased ECM density in AC contralateral to the trauma side exclusively in those animals that develop tinnitus. The effect is already visible 7 days after the initial acoustic trauma.

## Discussion

In this report, we have demonstrated that noise-induced tinnitus is associated with an increased ECM density in primary auditory cortex contralateral to the trauma side as early as seven days after the acoustic event. This result may point to trauma induced hemisphere-specific neuroplastic changes in the AC, where an ECM density increase may be an indication of tinnitus chronification.

The induction of a mild acoustic noise trauma—here monaurally—is a reliable method of inducing cortical plasticity (e.g., [24]) and tinnitus-related behavioral changes in our rodent animal model (e.g., [17, 25, 66]). To ensure an objective analysis of the data, only automated and parameter-free evaluation methods were used [49] to rule out human evaluation bias [68]. With a mean HL of 10 dB to 12 dB in the exposed ear (12 dB to 17 dB in the most affected frequency region between 2 and 8 kHz) four days after the trauma, the effect of the trauma applied here was comparably mild when compared to other studies (e.g., [66]). This fact could also explain the relatively low percentage of animals with behavioral signs of tinnitus (8/22, 36%). These behavioral signs of tinnitus were determined with a well-established statistical method [51] but are no proof that the animals really perceive a human-like tinnitus percept [10]. On the other hand, the animals with behavioral signs of tinnitus tend to show less hearing loss compared to animals without these behavioral signs. This is the case in the 13 days group and is always cross-validated with further physiological markers (e.g., [64]). The animal research results are generally in line with reports of differences in human tinnitus patients compared to non-tinnitus patients with hearing loss (e.g., [16]).

The ECM itself occupies between 10 and 20% of the entire brain volume and plays an important role in genesis, plasticity and regeneration of the central nervous system [6]. Furthermore, it is known that it plays a significant part in physiological as well as pathological conditions [33, 67] and has an important role in early fear conditioning [3], which could be associated with tinnitus, at least in humans [1]. The ECM can be divided into different subtypes: perineuronal nets and perisynaptic extracellular matrix [6]. Specifically for Mongolian Gerbils it has been shown that the intact ECM in its entirety is important for the correct function of the cortical layer dynamics and cross-columnar frequency integration of the auditory cortex [12] but its density modulation (i.e., removing it) can also be used for inducing neuronal plasticity and re-learning [21]. Furthermore, it has been shown that the ECM in the auditory cortex undergoes initial down- and long term upregulation during memory consolation over the course of 12 days [42], which fits quite well the timeframe of 13 days used in this study. Depending on the focus of different studies, cortical ECM density or its interaction with neurons or glia cells has been assessed with several different methods. Some studies used the ECM, e.g., as a marker for specific cell types [14, 41], which allowed them to count these cells within a specific region of the cortex. Other studies used the ECM signal as a more general indicator of, e.g., neuronal plasticity or the lack of it due to diseases [7, 21, 35]. In this study, we followed the latter methodology.

With the knowledge of the ECM being one important factor for indication of neuronal plasticity (maladaptive plasticity in the case of tinnitus), we investigated the ECM density of the auditory cortices of Mongolian gerbils at two timepoints after acoustic noise or sham exposure by using the described specific ECM affine marker (WFA-FITC). This marker is a lectin that specifically binds to chondroitin sulfates and thus labels chondroitin sulfate proteoglycans within the ECM [5, 47]. The fluorescence luminance of the marker is a direct measurement of the ECM density. To correct for possible differences in marker efficacy on the different histological slices, we corrected the luminance by relating it to a reference region in the brainstem. The 7 and 13 days after trauma were selected as timepoints, where a possible ECM rearrangement is beginning and finalized [21], respectively. Therefore, the neurophysiological changes in the auditory cortex—measured by electrophysiological recordings—leading to a chronification of the tinnitus percept should be observable in their development and completion [2, 65]. We found the clear evidence that animals with behavioral signs of tinnitus have a much more solidified ECM surrounding of their auditory cortex neurons contralateral to the trauma side than animals without these signs or healthy control animals already 7 days after the trauma. Especially the early results were somewhat unexpected in their strength and hardly different from the later effects. Those results are in line with the above-mentioned study of auditory learning and memory consolidation with an increase in ECM density after 12 days of continuous learning [42]. At both timepoints, the described effect after tinnitus induction by an acoustic trauma seems to be stronger in the cortical hemisphere contra-lateral to the trauma ear, which can be explained by the functional crossing of excitatory projections within the auditory pathway to the contra-lateral side. On the other hand, studies in mice did not show a significant increase in perineuronal net density over the course of up to 30 days after hearing loss (e.g., [41]). This could have several reasons. First, it could be that the induced hearing loss was much more severe (up to 49 dB at day one after trauma) than in our and other studies. This could lead to different responses in the auditory pathway and might not induce tinnitus at all [11]. In line with this view, we also did not find an increase of ECM density in NT animals. Second, a significant increase of ECM density is only found in the contra-lateral hemisphere of T animals, when combining both hemispheres—especially with low animal counts and low number of T animals—no significant increase may be found. Therefore, for interpretation of the data and new insight into the function of the auditory cortex it is crucial to compare only studies that at least roughly use the same methodology.

From the here described effect of the ECM being more solidified in tinnitus animals compared to control or non-tinnitus animals, several conclusions can be drawn regarding potential mechanistic tinnitus models, respectively. As it is not possible to provide a complete overview of all existing tinnitus models, we here focus on the most common ones.

First, the effect points to the assumption that in contrast to former models, chronic tinnitus is not a result of a re-organization of the tonotopic map after a cochlear damage as claimed by Mühlnickel et al. [39], but is instead characterized by a fixation of the wiring scheme after transient re-wiring in the cortex [2]. Indeed, the hypothesis raised by Mühlnickel et al. has been falsified by several other groups in various recent studies, where huge human tinnitus cohorts were investigated with fMRI [26, 28].

Second, our findings suggest that already during the early phase of tinnitus chronification, the responses of the neural system enter a neural attractor, which is possibly further reinforced by ongoing stiffening of the ECM in the auditory cortex (i.e., higher ECM density) over time. Very early effects have been shown for auditory learning [42, 45] with an indication of a downregulation in those early stages of the condition tested. At this moment, the exact time course of the tinnitus dependent ECM density changes have to remain speculative, as it is already present at our first measurment.

Nevertheless, the combined stochastic-resonance-predictive-coding model introduced by Schilling and co-workers might provide further insights [53] into this question. In a nutshell, the model consists of two parts. The brainstem part of the model (Erlangen model of tinnitus development, [57]) describes tinnitus as a result of intrinsically generated neural noise coming from outside the auditory system (see also [27, 69]) used to compensate for reduced hearing thresholds resulting from decreased cochlear output due to noise-induced cochlear damage [16, 29–32, 50, 57, 64].

For the cortical part of the model it was assumed that, first, the intrinsically generated neural noise is amplified along the auditory pathway via central gain effects and is then transmitted to higher brain structures such as the thalamus and the auditory cortex in a bottom-up manner. Such changes have been described also by other groups in animals [38] and suggested in humans (e.g., [20, 36]). Second, complementary to this bottom-up information flow, top-down mechanisms seem to play a crucial role in tinnitus development and especially tinnitus manifestation [22, 59]. Thus, the brain—and especially the cortex—is assumed to operate as a prediction machine trying to predict the cause of certain signals transmitted via the auditory pathway. Following the idea of the model, the cortex makes a default prediction (predictor for the Bayesian brain formalism), which is silence under normal conditions. The product of predictor (silence) and the top-down neuronal signal (likelihood) are the actual percept. According to the combined stochastic-resonance-predictive-coding model which unifies the bottom-up and top-down mechanisms [53], the increased bottom-up neural activity (neural noise) caused by the stochastic resonance effect is misinterpreted as neuronal signal evoked by a real auditory stimulus. The noise thus leads to increased activity and might also cause a decreased sensory precision—i.e., higher mean and lower variance of the likelihood—as brain states with low activity become very unlikely due to the continuous neural noise. In other words, the neural noise leads to continuous mispredictions of the cortex about the incoming auditory signal. Thus, the cortex updates its predictions in a pathological manner and starts to manifest a continuous auditory input as default prediction (updated predictor), or attractor. This attractor could be manifested in increased local ECM density after the tinnitus percept becomes chronic.

On the other hand, it is assumed that certain plastic changes in the cortex could reduce the described sensory precision changes or even prevent it from happening at all and therefore reset / keep the default prediction to silence [22, 59]. The here described results support also this idea, as in animals without tinnitus at both investigated timepoints the ECM density is not increased, neuronal plasticity is therefore still possible. In tinnitus animals however, the new synaptic weights and the mispredictions (falsely updated predictor) were consolidated by the solidifying ECM.

In the context of the described findings and the underlying hypotheses, one could speculate about a possible clinical relevance for the treatment of tinnitus patients. The most successful tinnitus specific therapies for many patients are the ones that include “coping counselling” (e.g., [4, 37]). In other words helping the patients to learn to live with the condition and reduce anxiety and burden. Other therapies try to influence the neuronal processing of tinnitus on different levels of the auditory pathway (e.g., [44, 61, 63]) with different degree of success. One could speculate that with the results of this study, the latter approach—in combination with some kind of relearning strategy, that still has to be developed—could be successful, as learning should initially reduce the ECM density and then solidify it in the new desired stable state. A drawback of such strategies could be that this relearning would have to take place without the constant “wrong” input from the tinnitus network. Here, newly developed strategies to suppress the tinnitus percept temporarily [52, 63] could play a promising role.

In conclusion, we assume the tinnitus percept to be caused by increased neural noise in an initial bottom-up mechanism. The increased activity is then misinterpreted as auditory input and manifested via an updated predictor (attractor) of higher cortical areas. If the misprediction is manifested via an increased cortical ECM density, there is no possibility for the system anymore to change this wrong predictor, and consequently tinnitus is perceived chronically. Any therapeutic strategy would have to take this into account.

## Data Availability

All data can be obtained by request to the authors.

## Abbreviations

ABR: Auditory brainstem response
AC: Auditory cortex
ANOVA: Analysis of variance
C: Control group animals
DCN: Dorsal cochlear nucleus
ECM: Extracellular matrix
GPIAS: Gap prepulse inhibition of acoustic startle
HL: Hearing loss
NT: Non-tinnitus group animals
T: Tinnitus group animals
WFA-FITC: Wisteria floribunda lectin-fluoresceine-5-isothiocyanate
